# Advanced Therapy Medicinal Products for Rare Diseases: State of Play of Incentives Supporting Development in Europe

**DOI:** 10.3389/fmed.2017.00053

**Published:** 2017-05-16

**Authors:** Andreas M. Farkas, Segundo Mariz, Violeta Stoyanova-Beninska, Patrick Celis, Spiros Vamvakas, Kristina Larsson, Bruno Sepodes

**Affiliations:** ^1^Human Medicines Research and Development Support Division, Product Development Scientific Support Department, European Medicines Agency, London, UK; ^2^College ter Beoordeling van Geneesmiddelen, Utrecht, Netherlands; ^3^Committee of Orphan Medicinal Products, European Medicines Agency, London, UK; ^4^Universidade de Lisboa – Faculdade de Farmácia, Lisboa, Portugal

**Keywords:** European orphan medicines regulation, Committee for Orphan Medicinal Products, orphan designation, cell therapy, gene therapy, advanced therapy medicinal products

## Abstract

In 2008, the European Union introduced the Advanced Medicines Regulation aiming to improve regulation of advanced therapy medicinal products (ATMPs). We applied the ATMPs classification definitions in this Regulation to understand the link of this emerging group of medicinal products and the use of the Orphan Regulation. A total of 185 products that can be classified as ATMPs based on this Regulation have been submitted for orphan designation. Prior to its introduction in 2008, 4.5% of the products submitted for orphan designation met these criteria. This percentage went up to 15% after 2008. We analyzed several parameters associated with active ATMP ODDs focusing on sponsor type and EU-Member State origin, therapeutic area targeted, and ATMP classification [i.e., somatic cell therapy medicinal product, tissue-engineered product (TEP), or gene therapy medicinal product (GTMP)] and the use of regulatory services linked to incentives such as the use of protocol assistance (PA) and other Committees [Committee for Advanced Therapies (CAT) and the Pediatric Committee]. The aim here was to gain insight on the use of different services. The UK submits the largest number of ATMPs for ODD representing ~30% of the total to date. Few submissions have been received from central and Eastern European Member States as well as some of the larger Member States such as Germany (3.6%). ATMPs ODDs were primarily GTMPs (48.7%) and SCTMPs (43.3%). TEPs only represented 8% of all submissions for this medicinal class. This is different from non-ODDs ATMPs where GTMPs make only 20% of ATMPs. A total of 11.7% of ATMP ODDs had received formal CAT classification. A total of 29.8% of all orphan drug (OD) ATMPs requested PA. A total of 71.8% did not have an agreed pediatric investigation plan (PIP). Four products (Glybera one PA; Zalmoxis two; Holoclar one; Strimvelis three) have received a marketing authorization (MAA) and a 10-year market exclusivity. Strimvelis also completed their PIP, which was compliant and received the additional 2-year extension to their 10-year market exclusivity. One OD ATMP (Cerepro) received a negative opinion for MAA. The use of services linked to incentives offered by different legislations for ATMP ODDs is low, indicating a need for increasing awareness.

## Introduction

Over the last 4.5 years, the Committee for Orphan Medicinal Products (COMP) has noted an increase in the number of submissions for advanced therapy medicinal products (ATMPs) seeking orphan drug (OD) designations. Indeed some of these products have recently received marketing authorization (MAA), i.e., strimvelis (autologous CD34^+^ enriched cell 50 fraction that contains CD34^+^ cells transduced with a retroviral vector that encodes for the human ADA 51 cDNA sequence), Glybera (i.e., alipogene tiparvovec), Zalmoxis [i.e., allogeneic T cells genetically 52 modified with a retroviral vector encoding for a truncated form of the human low affinity nerve 53 growth factor receptor (ΔLNGFR)] and Cerepro [herpes simplex I virus thymidine kinase (HSV-TK 54 Mut2)], and Holoclar (*ex vivo* expanded autologous human corneal epithelial cells containing stem 55 cells).

The introduction of the Advanced Therapies Regulation in 2008 and its implementation may be associated with this increase as it coincides with the development of ATMPs within the context of rare diseases. ATMPs are becoming an emerging and expanding class of innovative medicinal products since the introduction of the Advanced Therapies Regulation in 2008, which potentially offer an alternative approach to traditional small molecule medicinal products (i.e., chemicals) or biologicals such as recombinant proteins and monoclonal antibodies. This trend is expected to continue since these products can offer a more specific and causal/targeted treatment of many rare diseases for which the specific underlying cause is known, e.g., a gene defect.

The Orphan Office of the European Medicines Agency has conducted a survey specifically targeting ATMPs and orphan designation. The aim of the survey was to obtain a better understanding of the type of ATMPs submitted for orphan designation and how the services offered by the different European Legislations, Committees, and incentives were being used. In addition, the origin of the sponsor in EU Member State was also considered.

The Orphan Medicines Regulation (EC) No. 141/2000 offers a specific designation associated with incentives for a medicinal product intended to treat, prevent, or diagnose a rare disease, upon request by a sponsor. Orphan designation can be obtained at any stage of development, but before the submission of the MAA^1^ (1). In Europe, a third of all submissions for initial orphan designation currently are with preclinical *in vivo* data and two thirds with preliminary clinical data (2). The COMP was established through this legislation. The COMP assesses and recommends designation of products that submit for orphan designation in conditions that meet the criteria established in the Regulation. The European Commission grants the designation and the 10-year marketing exclusivity based on the recommendations from the COMP. The committee also provides input on protocol assistance (PA) questions on significant benefit.

The Advanced Therapies Regulation (EC) No 1394/2007 (3) established the creation of the Committee for Advanced Therapies (CAT) in 2009. It is a multidisciplinary committee, gathering together some of the best available experts in Europe to assess the quality, safety, and efficacy of ATMPs and to follow scientific developments in the field.

The main responsibility of the CAT is to prepare a draft opinion on each ATMP application submitted to the European Medicines Agency, before the Committee for Medicinal Products for Human Use (CHMP) adopts a final opinion on the granting, variation, suspension, or revocation of a MAA for the medicine concerned.

Other responsibilities of the CAT are to participate in Agency procedures for the provision of scientific recommendations on the classification of ATMPs in accordance with Article 17 of Regulation (EC) No 1394/2007 (3). ATMPs can be classified by the CAT as somatic cell therapy medicinal product, tissue-engineered product (TEP), or gene therapy medicinal product (GTMP).^2^ It is also involved in the certification of quality and non-clinical data for small- and medium-sized enterprises (SMEs) developing ATMPs.

Providing input for the Scientific Advice Working Party (SAWP) on quality, toxicology, and clinical development criteria associated with ATMPs is an important role that the CAT plays as well and will cover submissions for ATMPs, which have an orphan designation from the COMP. By doing this, the CAT also fulfils in part a request from the CHMP for input on scientific matters associated with ATMPs.

In addition, when a sponsor meets the criteria for SME as established under Commission Regulation (EC) No 2049/2005 (4), they can apply for SME status at the EMA. This legislation offers incentives that help SMEs with ATMPs to obtain additional free regulatory guidance and support through the development phases, fee reductions for scientific advice, and easier access to the services offered by the EMA. By combining SME status and orphan medicinal designation for an ATMP, there is an accumulation of incentives that can significantly reduce the regulatory consulting costs in the development phase.

The purpose of this article is to provide an overview of the COMP/EMA experience with the recent increase in ATMPs submitted for orphan designation, which are under development. A total of 185 products that can be classified as ATMPs based on this new Regulation have been submitted for orphan designation since the introduction of the orphan legislation in 2000. This emerging group of medicinal products and the various legislations being used to support their development and licensing is an area that appears to be evolving quickly. Evaluation of ATMP submissions received for orphan designation offers the possibility to obtain some preliminary insight into which of these technologies are being developed and for which target indication. It also offers some preliminary data into how sponsors are using the different incentives and legislations available to them to support them in development. The potential outcomes of the efficient use of these incentives have yet to be determined as only five products have come for licensing of which four were successful.

## Materials and Methods

The study sample includes all medicinal products that were granted orphan designation between 2001 and April 2016; listed as “active” ODs by the European Commission on June 2016 (i.e., where the designation had not “expired” or been “withdrawn”); and that fell under the definition of ATMPs as defined by the Advanced Medicines Regulation. Where no formal ATMP classification by CAT was available or when the designation preceded the Regulation, the EMA Orphan Office applied the CAT classification system (see text footnote 2) to active designations in the ODD database. We also assessed the origin of the sponsor by Member State, type of the applicant, i.e., private person, academic, consultant, SME, SME consultant, or pharmaceutical company without SME status at EMA. The SME status was verified in the EMA SME database. The therapeutic area for the ATMP submitted was assessed according to the first level of the Anatomical Therapeutic Chemical (ATC) Classification System (5).

For PA, only scientific advices after initial ODD were considered, and the number of PAs (initial and follow-up PAs) given was retrieved from the EMA scientific advice database. Agreed pediatric investigation plans (PIP) or waivers were identified by using the EMA pediatric database (PedRA).

To describe the nature and source of ATMPs submitted to the COMP since the introduction of the Orphan Regulation, we performed an analysis of the current product- and sponsor-related characteristics of the OD that would qualify as ATMPs. We assessed the origin of the sponsor by Member State, and whether the applicant was a private person, academic, consultant, SMEs, SME consultant, or pharmaceutical companies without SME status at EMA.

## Results

### Number of ATMP in ODD Applications

A total of 185 ATMP ODD, including medicines that meet the criteria but were designated before the ATMP Regulation, have been granted since the introduction of the Orphan Regulation in 2000. We grouped the designation by period 2000–2008 and 2009–2016. This grouping was decided to coincide with the introduction of the Advanced Therapies Regulation in 2008 and its implementation in 2009. Before the implementation of the Regulation, it was found that only 4.5% of orphan designated products were ATMPs. After introduction of the legislation between 2009 and 2016, it was found that it was 15%. For both periods, the number reflects the total number of ODD products in the ODD database that would meet ATMP criteria irrespectively whether they already applied for CAT classification or not.

### Member States

**Figure 1:**
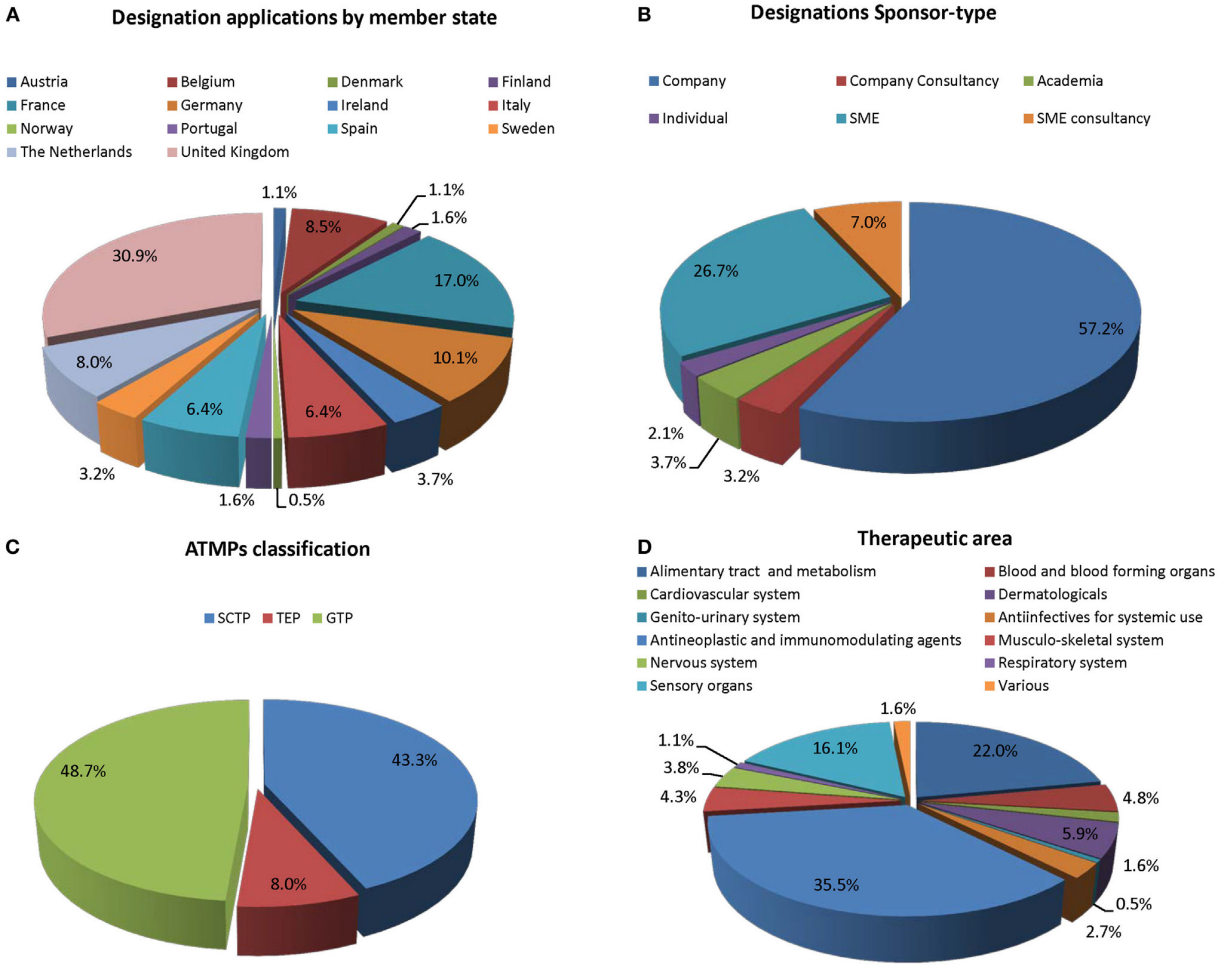
**The composition of active orphan drug designations since 2001 (*n* = 185)**. **(A)** ODD per Member State; **(B)** according to sponsor-type; **(C)** Committee for Advanced Therapies (CAT) classification; and **(D)** therapeutic area.

For the purpose of ODD, the sponsor’s legal representative has to be established within the EU, but can be a private person, a non-profit organization, or a commercial entity. It has been noted that most ATMP ODDs are submitted from a few Member States in Western Europe. However, these results should be viewed with the caveat that consultancies based in one of these Member States can obtain an ODD on behalf of a developer, thus the development might originate outside of the EU or be led in an EU Member State different than that of the sponsor holding the ODD (Figure 1A).

### Type of Sponsor

We found that 57.2% of the sponsors were pharmaceutical companies (Figure 1B). Among these, many could be considered SMEs but did not hold SME status. SMEs and SME consultancies represent 26.7 and 7%, respectively, of all ODD of ATMPs. Significantly lower submissions from academics (7.4%) and private individuals (4.2%) (Figure 1B).

### Therapeutic Areas According to ATC Classification

Of the 155 ATMPs with OD designation antineoplastic and immunomodulating agents represent the largest group (35.5%), followed by treatment of defects in sensory organs and metabolic disorders (22 and 16.1%, respectively) (Figure 1D). All other therapeutic areas are occasionally represented (below 0.5%) and represent 5.9% of overall OD ATMPs.

### By Type of ATMP

**Figure 2:**
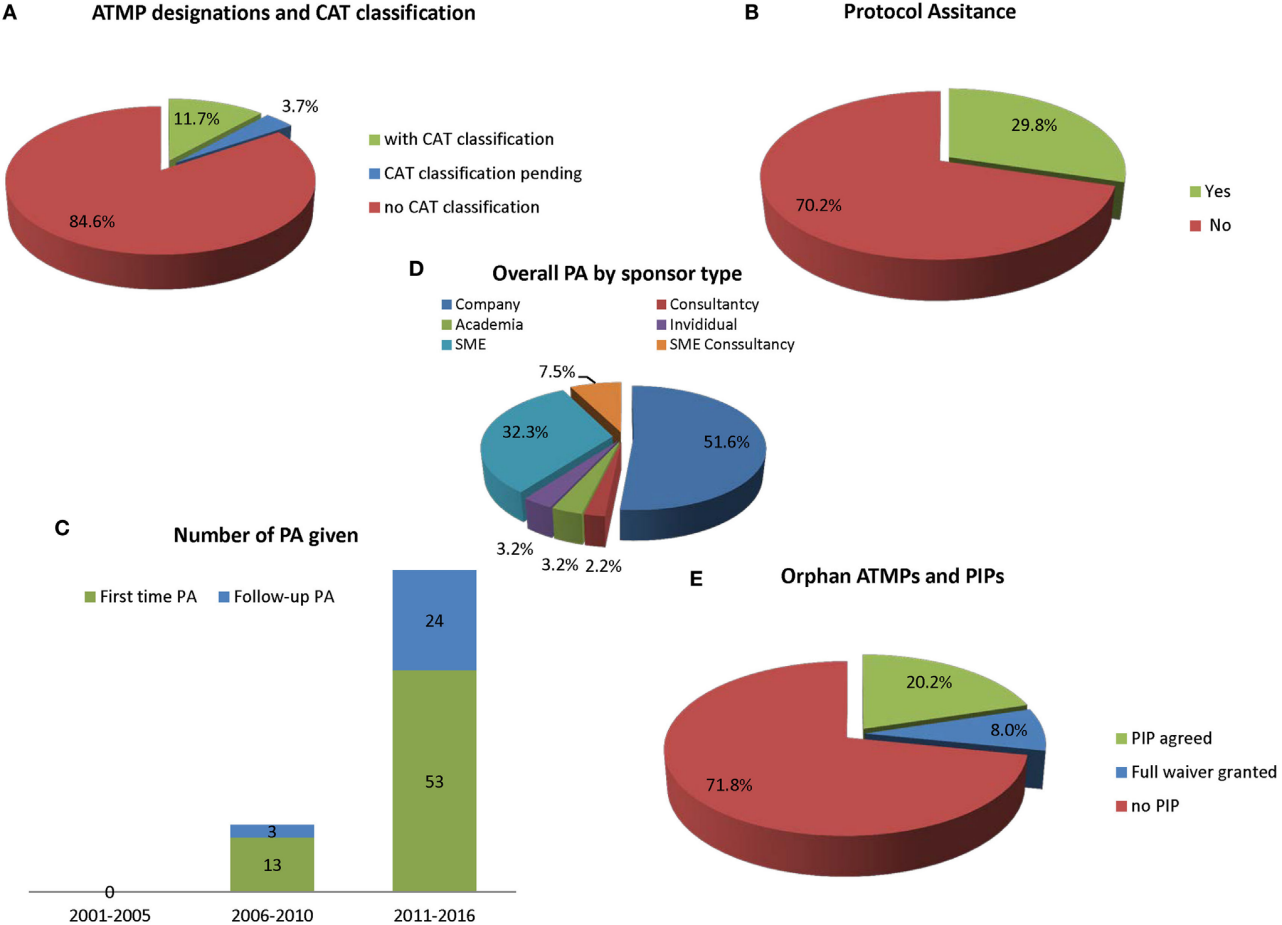
**The current use of advisory services by EMA (*n* = 185)**. **(A)** Advanced therapy medicinal product (ATMP) ODDs subdivided according to the percentage of official Committee for Advanced Therapies (CAT) classifications; **(B)** according to protocol assistance (PA) used for at least once; **(C)** use of PA usage over time also showing first-time contact and follow-up advices; **(D)** overall PA procedures given according to sponsor type; and **(E)** percentage of ATMP ODDs with an agreed pediatric investigation plan (PIP) of waiver.

Gene therapy medicinal products (48.7%) and SCTMPs (43.3%) represent similar percentages of the total number of designation, while TEPs in contrast only represents a smaller part (8%) (Figure 1C). Most medicines (84.6%) have not received classification (Figure 2A); for 3.7%, the classification was pending, and 11.7% had received formal classification.

### Protocol Assistance

It was noted that 29.8% of all OD ATMPs have requested scientific advice. This is known as PA when a product obtains orphan designation (Figure 2B). As shown in Figure 2C, there were no PAs given on medicines that could be considered ATMPs between 2001 and 2005. Between 2006 and 2010, there were 16 PA procedures including 3 follow-up advices for 13 ATMPs. Following introduction of the Advanced Medicines Regulation in 2007, an increase in PA was noted between 2011 and the first half of 2016, with a parallel increase in the number of orphan designations: 77 advices (including 24 follow-up PAs) were given for 53 ATMPs. There were no differences between the OD sponsor types requesting or not requesting PA for ATMPs (Figure 2D). Four products that came for PA (Glybera one PA; Zalmoxis two; Holoclar one; Strimvelis three) have received an MAA. One OD ATMP (Cerepro) that had not come for PA received a negative opinion at MA.

### Pediatric Investigation Plan

Figure 2E shows that 71.8% of all ATMPs with ODD do not yet have an agreed PIP. Thirty-eight orphan-designated ATMPs have a PIP (20.2% of the OD ATMPs have a PIP with 8.0% deferrals) and 15 have a waiver. For designated OD, completion of a PIP, which is compliant with the PIP decision and which leads to a modification of the summary of product characteristics (SmPC), can be rewarded by an extension of the orphan market exclusivity by 2 years (12 years in total).

### CAT Classification

We analyzed how many sponsors that had orphan designation for an ATMP had come for CAT classification. Overall it was noted that since the introduction of the Advanced Therapies Regulation, 11.7% of OD ATMPs had CAT classification and 3.7% were pending. A total of 84.6% of OD ATMPs did not have CAT classification (Figure 1A).

## Discussion

The purpose of this article is to provide an overview of the COMP/EMA experience with a recent increase in ATMPs submitted for orphan designation. A total of 185 products that can be classified as ATMPs based on the Advanced Therapies Regulation have been submitted for orphan designation since the introduction of the orphan legislation in 2000. This emerging group of medicinal products and the various legislations being used to support their development and licensing is an area that appears to be evolving quickly. Evaluation of ATMP submissions received for orphan designation offers the possibility to obtain some preliminary insight into which of these technologies are being developed and for which target indication in rare conditions. It also offers some preliminary data into how sponsors are using the different incentives and legislations available to them to support their development. The potential outcomes of the efficient use of these incentives has yet to be determined as only five products have come for licensing of which four were successful.

The increase in positive opinions (15% between 2009 and 2016 compared with only 4.5% between 2000 and 2008) after the introduction of the Advanced Therapies Regulation in 2008 cannot be ignored and points to an impact of the legislation, which needs further study. It is hypothesized that this may be due to recent reported breakthroughs in the development of new manufacturing processes and standards to make GMP production more feasible. In addition, the development of newer and safer viral vectors might have led to a revival of gene therapy approaches (6).

Among the OD ATMPs submitted, GTMPs represent the largest group closely followed by SCTMPs. TEPs only represent 8%. This is in clear contrast with the distribution of the overall CAT classification of ATMPs (including common diseases) where GTMPs make up roughly 20% of ATMPs, and the remaining are evenly distributed between TEPs and SCTMPs. The reasons for this difference could be due to the type of condition that is being targeted. It was noted in the data we analyzed that treatment of defects in sensory organs and metabolic disorders represented 22 and 16.1%, respectively. This might be due to the fact that ATMPs, in particular targeted gene therapy, hold potential as a therapeutic alternative in diseases are caused by a single gene defect. For example, there are 10 active OD ATMPs for retinitis pigmentosa. It was noted that the treatment of defects in sensory organs and metabolic disorders were two areas where there is a higher proportion of designations for OD ATMPs (2). The situation is rather different in oncology, which represents around another third of the ODs where there is complex underlying pathology.

It was noted that sponsors based in the UK submits the largest number of ATMPs for ODD representing ~30% of the total to date. There is of course a caveat in that many sponsors in the UK are consultancies holding OD ATMPs for non-EU sponsors many of whom are based in the United States. Very few submissions have been received from central and Eastern European Member States and unexpectedly from some of the larger Member States such as Germany (3.6%).

The CAT is consulted by CHMP and SAWP regarding ATMPs as has been discussed in Section “Introduction.” COMP interacts with CHMP within a similar context although this covers all medicinal products for rare diseases that have come for orphan designation. This opens the possibility for use of the different legislations within the context of the incentives they offer as well as the input they give for medicinal products. We analyzed the use of PA for OD ATMPs as it is known that both the COMP and the CAT can be consulted by SAWP/CHMP. It was noted that 29.8% of all OD ATMPs have requested scientific advice. It was also noted overall that since the introduction of the Advanced Therapies Regulation, only 11.7% of OD ATMPs have received CAT classification. The limited use of the classification as well as PA by sponsors holding an OD ATMP appears to indicate a limited awareness of what the CAT does as well as the SAWP. It was also noted that 20.2% of the OD ATMPs have obtained a PIP with 8.0% deferrals. Use of the Paediatric Development Committee, which is an obligation for new active substances, appears also to be underutilized. Sponsors of ATMPs appear to need to be more aware of the need for a PIP as they could be blocked at the stage of validation. In addition, the additional reward of +2-year marketing exclusivity should they have a completed compliant PIP, which can then be used to change the SmPC when the data are submitted to CHMP. It should be noted that often PIPs for ATMPs are deferred due to safety concerns. Although we do not have specific data regarding the use of incentives for sponsors who are SMEs with OD ATMPs, it should be note that 27% of the 185 designations are SMEs.

There is an additional reward of a 2-year extension added to the 10-year market exclusivity if a sponsor completes the pediatric development in compliance with an agreed PIP. Submission and agreement of a PIP, a deferral, or a waiver is free of charge but is required before MAA submission if a product is considered a new active substance. Although presubmission discussions are voluntary, the EMA strongly encourages sponsors to get in contact with the Paediatric Office as many orphan conditions also affect the pediatric population, and it is mandatory to have a PIP at the time of submission for MAA (7). To date, none of the limited number of ATMPs, which have an agreed compliant PIP and have obtained a MAA, have obtained the 2-year pediatric extension.

It should be noted that the Advanced-Therapy Medicines Regulation came into force after 2009, which shortly followed the introduction of the Paediatric Regulation after 2006. PIPs may take some time before they are completed, especially if the trials are modified if the condition does not affect children, the sponsor may obtain a waiver. Four ATMPs with orphan designation (Glybera, Strimvelis, Zalmoxis, and Holoclar) have been successful in obtaining a MAA and obtaining the 10-year market exclusivity. Three had ongoing PIPs at the time of submission for MAA. One, namely Strimvelis, has obtained the 2-year extension of the market exclusivity.

In this article, we have highlighted the importance of the emerging field of ATMPs with orphan designation. There are a high number of sponsors for ATMPs localized in some EU countries. In these countries, the awareness of the orphan designation appears to be high. GTMPs appear to be of highest interest in rare diseases. This could be due to the possibility they offer to treat certain rare diseases, in particular those caused by single gene defects. As a consequence, an increase in requests for services offered by the European Medicines Agency could be anticipated. There is a need for greater cooperation between sponsors and regulators on the development of OD ATMPs to support successful outcomes.

Obtaining ATMP classification by the CAT is viewed by the EMA as an excellent opportunity for early interaction and to get valuable regulatory input, as is PA, which only few sponsors of ATMPs have requested. Early and recurrent communication between sponsors and the Agency throughout development should facilitate the MAA process.

## Author Contributions

The data collection and analysis was done by AF under the supervision of SM. All other authors contributed to interpretation of the data, drafting, and editing the manuscript.

## Conflict of Interest Statement

The authors declare that the research was conducted in the absence of any commercial or financial relationships that could be construed as a potential conflict of interest. The views expressed in this article are the personal views of the authors and may not be understood or quoted as being made on behalf of or reflecting the position of the European Medicines Agency or one of its committees or working parties.

## Footnotes

^1^http://ec.europa.eu/health/files/orphanmp/2014-03_guideline_rev4_final.pdf.

^2^http://www.ema.europa.eu/docs/en_GB/document_library/Scientific_guideline/2015/06/WC500187744.pdf.
